# Zero tolerance for healthcare-associated MRSA bacteraemia: is it realistic?

**DOI:** 10.1093/jac/dku128

**Published:** 2014-04-30

**Authors:** M. Estée Török, Simon R. Harris, Edward J. P. Cartwright, Kathy E. Raven, Nicholas M. Brown, Michael E. D. Allison, Daniel Greaves, Michael A. Quail, Direk Limmathurotsakul, Matthew T. G. Holden, Julian Parkhill, Sharon J. Peacock

**Affiliations:** 1 Department of Medicine, University of Cambridge, Cambridge, UK; 2 Department of Microbiology, Cambridge University Hospitals NHS Foundation Trust, Cambridge, UK; 3 Public Health England, Clinical Microbiology and Public Health Laboratory, Cambridge, UK; 4 Wellcome Trust Sanger Institute, Hinxton, Cambridge, UK; 5 Department of Medicine, Cambridge University Hospitals NHS Foundation Trust, Cambridge, UK; 6 Mahidol-Oxford Tropical Medicine Research Unit, Mahidol University, Bangkok, Thailand

**Keywords:** methicillin-resistant *Staphylococcus aureus*, outbreak, whole-genome sequencing

## Abstract

**Background:**

The term ‘zero tolerance’ has recently been applied to healthcare-associated infections, implying that such events are always preventable. This may not be the case for healthcare-associated infections such as methicillin-resistant *Staphylococcus aureus* (MRSA) bacteraemia.

**Methods:**

We combined information from an epidemiological investigation and bacterial whole-genome sequencing to evaluate a cluster of five MRSA bacteraemia episodes in four patients in a specialist hepatology unit.

**Results:**

The five MRSA bacteraemia isolates were highly related by multilocus sequence type (ST) (four isolates were ST22 and one isolate was a single-locus variant, ST2046). Whole-genome sequencing demonstrated unequivocally that the bacteraemia cases were unrelated. Placing the MRSA bacteraemia isolates within a local and global phylogenetic tree of MRSA ST22 genomes demonstrated that the five bacteraemia isolates were highly diverse. This was consistent with the acquisition and importation of MRSA from the wider referral network. Analysis of MRSA carriage and disease in patients within the hepatology service demonstrated a higher risk of both initial MRSA acquisition compared with the nephrology service and a higher risk of progression from MRSA carriage to bacteraemia, compared with patients in nephrology or geriatric services. A root cause analysis failed to reveal any mechanism by which three of five MRSA bacteraemia episodes could have been prevented.

**Conclusions:**

This study illustrates the complex nature of MRSA carriage and bacteraemia in patients in a specialized hepatology unit. Despite numerous ongoing interventions to prevent MRSA bacteraemia in healthcare settings, these are unlikely to result in a zero incidence in referral centres that treat highly complex patients.

## Introduction

Reducing healthcare-associated infections caused by methicillin-resistant *Staphylococcus aureus* (MRSA) represents an important healthcare priority.^1,2^ Evidence that this can be achieved includes an 87% reduction in MRSA bacteraemias reported to Public Health England from 7291 in 2001/02 to 924 in 2012/13.^3^ This followed the introduction of mandatory surveillance for *S. aureus* bacteraemia, together with the implementation of a package of infection control measures including hand hygiene, MRSA screening and decolonization, patient isolation and infection prevention care bundles.^1,2,4–8^ England has now entered a new phase of control in which the term ‘zero tolerance’ has been used with reference to healthcare-associated infections, including MRSA bacteraemia.^8,9^

‘Zero tolerance’ was first used to describe policing techniques in New York City in situations relating to criminal acts, often resulting in punishment for minor infringements and not taking into account extenuating circumstances.^10^ This approach can be equated with several types of healthcare event such as wrong site surgery or retained surgical instruments, which result from a single act of human error that can be prevented through modification of practice. However, zero tolerance of healthcare-associated infections such as MRSA bacteraemia is likely to be more difficult to achieve because of the complex and multifactorial processes underlying MRSA acquisition and subsequent infection, including host factors, staff training and behaviour, the physical environment and patient movement through the healthcare network. The drive to reduce MRSA infections to zero is also hindered by the increasing number of patients who are elderly or have reduced immunity to infection.

Cambridge University Hospitals NHS Foundation Trust (CUH) is a large university teaching hospital in England where MRSA bacteraemia rates have fallen in parallel with the national trend. Between September 2011 and August 2012, five MRSA bloodstream infections occurred in four patients admitted to the hepatology ward. This triggered a detailed clinical and epidemiological investigation, which was supported by microbial sequencing in order to better understand the basis for the cluster and to examine whether these cases were potentially avoidable. Our investigations excluded an outbreak on the hepatology ward and highlighted several key barriers to the prevention of MRSA bacteraemia in this population, including pre-existing MRSA carriage at the time of presentation, failure of MRSA decolonization therapy and high risk for MRSA carriage and the progression from MRSA carriage to disease. These findings add to the limited information available on MRSA carriage and infection in patients with cirrhosis.^11–16^

## Methods

### Study setting and participants

CUH is a secondary and tertiary referral hospital in England with 1000 beds and 67 000 inpatient admissions a year. All patients admitted to CUH are screened for MRSA carriage and those in critical care units undergo weekly MRSA screening. Infection control management structures, standards, policies and procedures supporting the prevention and control of infection at CUH are described in the Infection Control Annual Report.^17^ The hepatology service at CUH is a specialist liver unit that provides tertiary care for advanced acute and chronic liver and biliary diseases and liver transplantation. This service has >7800 outpatient attendances, nearly 2000 elective admissions and ∼300 emergency admissions per year referred from Eastern England and the East Midlands. In 2012, ∼180 patients were assessed for liver transplantation and 75–85 liver transplants are performed each year. At the time of this study, routine screening for MRSA carriage on the hepatology ward was only performed on the day of admission.

### Bacterial isolation, antimicrobial susceptibility testing, DNA sequencing and analysis

Clinical specimens taken during routine clinical care were processed and stored at the Clinical Microbiology and Public Health Laboratory at CUH. MRSA was identified using MRSA Brilliance agar (Oxoid, Basingstoke, UK) and a PBP2a latex agglutination test (Mastalex, Mast Diagnostics, Bootle, UK). Antimicrobial susceptibility testing was performed using the AST-P620 card on the Vitek 2 system (bioMérieux, Marcy-l'Étoile, France). MRSA isolates were assigned a unique identification code prior to DNA extraction and library preparation, which was performed as previously described.^18^ DNA libraries were sequenced using the Illumina MiSeq platform (Illumina Inc.) to generate 150 bp paired-end reads. Sequence reads were aligned to the chromosome and plasmid (accession numbers HE681097 and CP002148, respectively) of a reference MRSA genome [HO 5096 0412, sequence type (ST) 22] to identify single-nucleotide polymorphisms (SNPs). Bioinformatic analyses were performed by an investigator who was blinded to the clinical and epidemiological data. We used SNPs in the core genome for the phylogenetic analysis, excluding variation in the accessory genome that may have arisen through horizontal gene transfer from unrelated lineages. Sequence data have been deposited in the European Nucleotide Archive (Table S1, available as Supplementary data at *JAC* Online).

### Ethics

Ethics approval was not required as the study was conducted as part of surveillance and management of healthcare-associated infection. Approval for the sequencing of bacterial isolates was obtained from the CUH Research and Development Department.

### Statistical analyses

A hospital dataset containing all patients admitted to CUH between 1 January 2003 and 31 December 2012 (*n* = 420 949) was combined with a microbiology database containing the MRSA results of all individuals. Statistical analyses were performed using STATA version 12.0 software (StataCorp, College Station, TX, USA), including calculation of incidence rates of MRSA bacteraemia and the use of Poisson regression to calculate the incidence rate ratio (IRR). The rate at which patients had their first documented episode of MRSA carriage at CUH and the rate of progression from MRSA carriage to bacteraemia were calculated per 100 000 admission days for three medical specialities (hepatology, nephrology and geratology) and all other specialities combined.

## Results

### Clinical and bacterial genomic investigation of four patients with MRSA bacteraemia

**Figure 1.**
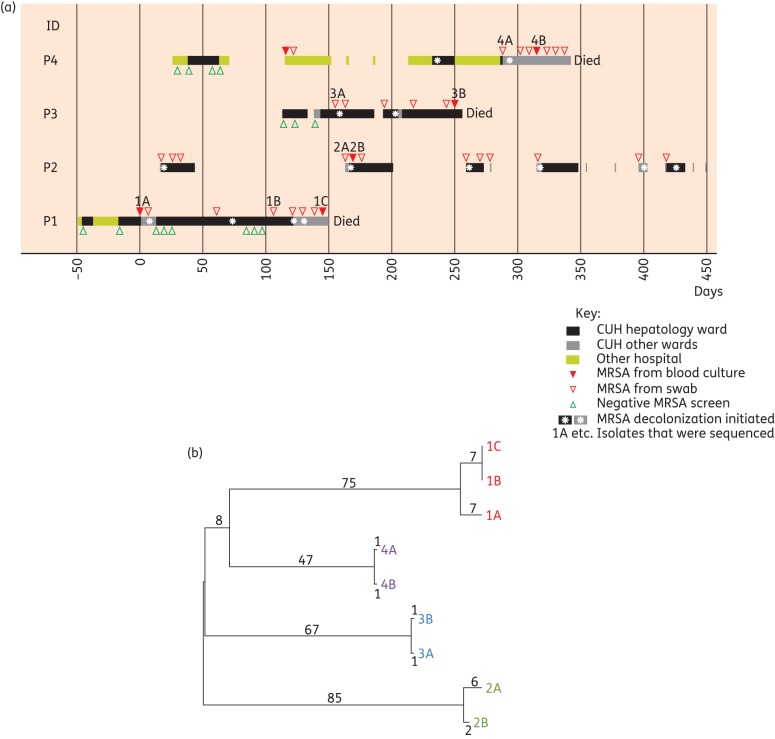
Epidemiology and bacterial phylogeny of MRSA bacteraemia cases. (a) Epidemiological map of four patients (P1–P4) with MRSA bacteraemia. Each row represents a single patient and the coloured blocks for each represent the time spent as an inpatient on the hepatology ward, other wards at the CUH or at other hospitals. The length of each box denotes the duration of admission and the scale bar represents days after the date of the first bacteraemia. (b) Phylogenetic tree based on whole-genome sequencing of nine carriage and bacteraemia isolates of MRSA from the four patients shown in (a). The numbers shown represent SNP differences between isolates.

Five MRSA bacteraemias occurred in four patients admitted to the hepatology ward at CUH between September 2011 and August 2012. An epidemiological investigation demonstrated varying degrees of overlap in admission to the ward for these cases (Figure 1a). All four patients had decompensated chronic liver disease, two had been referred for liver transplant assessment and one had hepato-renal syndrome (Table 1). One patient (P1) was MRSA screen negative on admission but developed two episodes of MRSA bacteraemia just >4 months apart at CUH. Three patients (P2, P3 and P4) were known to be colonized with MRSA prior to admission, one of whom (P4) had had a previous MRSA bacteraemia 7 months earlier at another nearby hospital. The antibiotic susceptibility profiles for the MRSA bacteraemia isolates from three patients (P1, P2 and P4) were identical (resistance to cefoxitin, oxacillin and ciprofloxacin) and showed two antibiotic susceptibility differences from the isolate from P3, which was additionally resistant to erythromycin, with inducible resistance to clindamycin.

**Table 1.** Clinical features, risk factors for MRSA carriage/infection and outcome in patients with MRSA bacteraemia

Patient	Clinical presentation	Risk factors for MRSA	Root cause analysis	Outcome
P1	decompensated cirrhosis, right pleural effusion, oedema, cellulitis	previous hospital admission; ciprofloxacin prophylaxis; intravascular catheter; skin and soft tissue infection	first bacteraemia secondary to cellulitis; second bacteraemia secondary to intravascular catheter	died
P2	decompensated cirrhosis, tense ascites, paraumbilical hernia with superficial ulceration, oedema	previous hospital admission; previous MRSA colonization; ciprofloxacin prophylaxis; ulcerated skin; intravascular catheter	MRSA bacteraemia secondary to skin ulceration	survived
P3	decompensated cirrhosis and infected leg ulcers, gastrointestinal haemorrhage	previous hospital admission; previous MRSA colonization; ciprofloxacin prophylaxis; chronic leg ulcers; intravascular catheter	MRSA bacteraemia secondary to infected leg ulcers	died
P4	decompensated cirrhosis, encephalopathy, ascites, hepato-renal syndrome	previous hospital admission; previous MRSA colonization and bacteraemia; ciprofloxacin prophylaxis; intravascular catheters; urinary catheter	MRSA bacteraemia secondary to intravascular catheter	died

Nine MRSA isolates comprising five from blood (1A, 1C, 2B, 3B and 4B) and the first stored non-bacteraemic isolate from each patient (1B, 2A, 3A and 4A) (Figure 1a) were analysed by whole-genome sequencing. Isolates from three patients (P1, P3 and P4) were found to be multilocus ST22 (which accounts for ∼80% of hospital-acquired MRSA bacteraemias in the UK) while isolates from P2 were of ST2046, a single-locus variant of ST22. Pairwise whole-genome comparisons indicated that each patient was infected by their own carriage isolate (Figure 1b). In contrast, phylogenetic analysis showed that the MRSA isolates from the four patients were genetically diverse, with between 122 and 168 core SNPs separating the most recent common ancestral sequences of the four patients (Figure 1b). Based on a previously calculated substitution rate of 1.3 × 10^−6^ (95% highest posterior density 1.2 × 10^−6^ to 1.4 × 10^−6^) substitutions per site per year for ST22,^19^ such levels of diversity would represent between 32 and 53 years since the clones infecting any pair of patients shared a common ancestor. We concluded that despite the epidemiological links between the four patients, the five bacteraemias were independent events rather than related to a protracted outbreak on the ward.

### MRSA is largely imported into the hepatology ward

**Figure 2.**
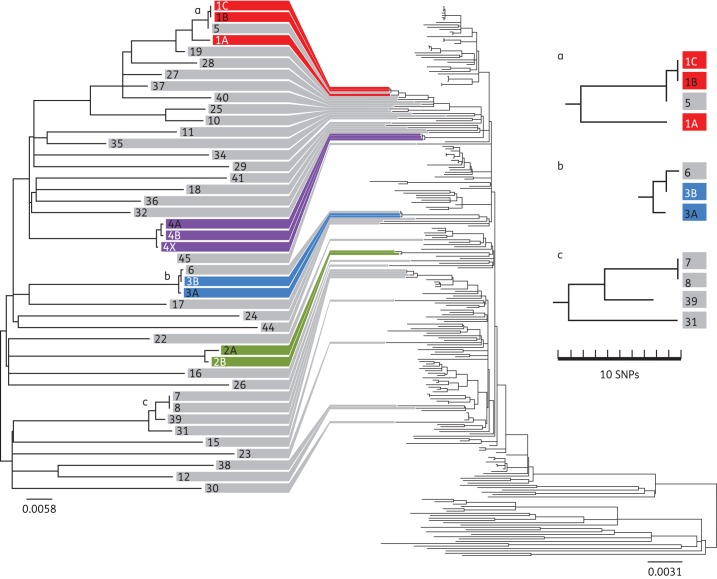
Phylogenetic context of MRSA isolates from hepatology patients. Phylogenetic tree based on the whole-genome sequence of nine MRSA isolates from four bacteraemia patients (P1–P4, different colours) and 42 MRSA isolates from patients with recent MRSA acquisition admitted to the hepatology ward (P5–P47, coloured grey, left-hand tree). The same isolates were included in a phylogenetic tree with the 193 ST22 MRSA isolates from a global collection (right-hand tree).^19^ Grey lines linking the two trees indicate the position of the hepatology isolates in the global phylogeny. The three clusters (a, b and c) represent suspected MRSA transmission events, two of which were corroborated by epidemiological evidence.

The genetic distance between the MRSA isolates from the four study patients led us to hypothesize that the MRSA carried by this patient population may have been acquired at other hospitals in the referral network, rather than on the hepatology ward at CUH. To investigate this, the microbiology laboratory database was used to identify patients who were found to be MRSA positive for the first time at CUH between September 2011 and August 2012 and who had been admitted to the hepatology ward in the year prior to their first positive sample. Forty-two patients who had at least one MRSA isolate available for whole-genome sequencing were identified. A phylogenetic tree was constructed for these 42 isolates, the 9 isolates from the bacteraemia cases described above and 1 bloodstream isolate from P4 who had an earlier bacteraemia at a neighbouring hospital (isolate 4X; Figure 2). To put these local data into a global context, we also constructed a tree including 193 ST22 MRSA isolates collected from 15 countries between 1990 and 2009 that had been sequenced previously (Figure 2).^19^ The 9 isolates from the bacteraemia cases and the 42 carriage isolates were highly diverse and scattered throughout the global ST22 tree. This is consistent with MRSA being largely imported into, rather than acquired on, the hepatology ward. Further support for this conclusion is provided by an additional isolate from P4 from a previous bacteraemia episode (isolate 4X), which occurred 7 months earlier at a different hospital and was only three SNPs distant from the common ancestral sequence of the isolates from the same patient taken at CUH. This isolate was 72 SNPs away from any other MRSA isolate at CUH, indicating that this MRSA isolate was imported with the patient.

Genetic evidence of MRSA transmission between the 46 patients (4 bacteraemia and 42 non-bacteraemia patients) on the hepatology ward was sought. This approach detected three possible MRSA transmission events. The first involved P1 and an individual with MRSA carriage (P5), whose admission on the hepatology ward overlapped for 2 weeks. The epidemiological data suggested that P1 acted as the donor. This is consistent with the phylogenetic analysis, in that the genetic distance between the first and second bacteraemia isolates from P1 (4 months apart) was 15 SNPs, compared with 1 SNP between the second P1 bacteraemia isolate and the non-bacteraemia isolate from P5 (Figure 2, cluster a). The second event occurred 3 months later and involved P3 and an individual with MRSA carriage (P6) who overlapped on the hepatology ward for 17 days (Figure 2, cluster b); the epidemiological data suggested that P3 acted as the donor. A third putative transmission event based on genomic evidence (two MRSA isolates with a single SNP difference; Figure 2, cluster c) was not corroborated by epidemiological data, with no overlap or link found for the two patients involved (P7 and P8). Possible explanations for this include acquisition of MRSA by both patients from a third patient or a staff member or transmission in another setting, e.g. the outpatient clinic or at another hospital. Overall, the picture drawn by the genomic evidence is more consistent with acquisition across the large catchment area for patients referred to the hepatology service, rather than an extended transmission network on the hepatology ward at CUH.

### Patients with chronic liver disease are highly vulnerable to MRSA carriage and bacteraemia

Chronic liver disease is associated with an increased risk of bacterial infections. This may be present on admission or develop during hospitalization in 30% of patients with cirrhosis and is associated with a 4-fold increase in mortality.^11,20^ Although chronic liver disease is not a widely recognized risk factor for MRSA carriage or bacteraemia,^21^ there is evidence to suggest that patients with cirrhosis may be at increased risk of developing MRSA infection.^12–14^ To investigate this in our setting, we calculated the incidence rate of first documented MRSA carriage at CUH (per 100 000 admission days at risk) for three medical specialties (hepatology, nephrology and geratology) and all other specialties combined (which was taken as the background rate) between 1 January 2003 and 31 December 2012 using a univariable Poisson regression model (Table 2). The incidence rate of newly documented MRSA carriage in the hepatology group was more than two and a half times that of the background rate (IRR 2.6, 95% CI 2.3–2.9) and was second only to the geriatric group (IRR 3.2, 95% CI 3.0–3.4). The rate of progression from MRSA carriage to bacteraemia was examined to see if it differed between these patient groups. The incidence rate of developing MRSA bacteraemia in the hepatology group was more than twice that of the background rate (IRR 2.1, 95% CI 1.6–2.7). In contrast, the incidence rate in the geriatric group was lower than that of the background rate (IRR 0.5, 95% CI 0.3–0.6), indicating that this patient group was less likely to progress from MRSA carriage to bacteraemia.

**Table 2.** Incidence rate of MRSA carriage acquisition and of progression from MRSA carriage to MRSA bacteraemia, according to speciality at CUH between 1 January 2003 and 31 December 2012

Speciality	Total number of cases with new MRSA carriage acquisition	Incidence rate of MRSA carriage acquisition (per 100 000 patient days at risk)	IRR (95% CI)	*P* value
All other specialities	4282	155	1.0	<0.0001
Hepatology	363	368	2.6 (2.3–2.9)	
Nephrology	133	82	0.6 (0.5–0.7)	
Geriatrics	1329	446	3.2 (3.0–3.4)	
	Total number of cases who progressed from MRSA carriage to bacteraemia	Incidence rate of developing MRSA bacteraemia (per 100 000 patients with MRSA carriage days)	IRR (95% CI)	*P* value
All other specialities	342	213	1.0	<0.0001
Hepatology	60	430	2.1 (1.6–2.7)	
Nephrology	24	270	1.2 (0.8–1.8)	
Geriatrics	59	99	0.5 (0.3–0.6)	

### Preventing MRSA bacteraemia in MRSA carriers

Having established that MRSA acquisition often occurs prior to admission to the hepatology ward and that patients with liver disease are at high risk of MRSA acquisition and developing MRSA bacteraemia, the actions that might have been taken to prevent progression from carriage to bloodstream infection were considered. In the UK, it is standard practice to decolonize patients who are MRSA positive using a regimen of mupirocin (Bactroban) 2% nasal ointment and topical antisepsis (Octenisan wash at CUH).^22^ A retrospective case notes review revealed that all four bacteraemia patients had received MRSA decolonization therapy prior to the development of bacteraemia (Figure 1). This was universally unsuccessful and may have failed because of the presence of an intravascular catheter (*n* = 4), skin ulcers (*n* = 2), skin and soft tissue infection (*n* = 1) or a urinary catheter (*n* = 1). A root cause analysis was conducted for each of the five episodes of MRSA bacteraemia. This concluded that three episodes were associated with skin conditions (which were considered unavoidable) and two were attributable to intravascular catheters. The latter were essential for the clinical management of the patients and no line care issues were identified during the root cause analyses. These cases highlight the challenges in preventing MRSA infection in MRSA-colonized patients presenting with end-stage liver disease.

## Discussion

We investigated a cluster of MRSA bacteraemias in our hospital to define contributory factors that we perceived were under our control and to distinguish these from factors that were not. A striking finding was that MRSA associated with carriage in patients who had at least one admission to the hepatology ward were highly genetically diverse, with a diversity similar to that of a global collection of ST22 MRSA isolates spanning a 20 year period. This strongly suggests that MRSA is frequently imported into the ward from multiple independent sources, rather than spreading on the ward, and is likely to be related to acquisition within the extensive referral network for this specialist service. Furthermore, the SNP distances between the isolates associated with the cluster of bacteraemias precluded the possibility of an outbreak having occurred on the ward.

We found that the rate of MRSA colonization and infection in hepatology patients was high compared with other patient groups, suggesting that cirrhosis is a risk factor for both MRSA colonization and disease. A previous study of 84 patients with cirrhosis admitted to a liver transplant unit in the USA found that MRSA carriage occurred in 29% of patients and was associated with more severe liver disease and an increased risk of developing *S. aureus* infections.^15^ Central venous catheter use was independently associated with *S. aureus* infections in carriers and MRSA carriers had a higher mortality than non-carriers. Another study by Campillo *et al*.^12^ examined the epidemiology of hospital-acquired infections in cirrhotic patients and found an MRSA carriage prevalence of 16.7% and a 10-fold increased risk of MRSA infections in carriers. In-hospital mortality was higher in MRSA carriers than non-carriers and MRSA carriage and bacteraemia were independently associated with death. A second study by Campillo *et al*.^13^ examined episodes of spontaneous bacterial peritonitis and bacteraemia in 200 patients with cirrhosis. The prevalence of MRSA was 24.8% and MRSA infections were more likely to recur and present at sites other than the bloodstream and ascites. Older age, advanced liver disease and staphylococcal infections were independently associated with death. A more recent study examined *S. aureus* bacteraemia in patients with and without cirrhosis.^16^ Patients with cirrhosis had a higher 30 day mortality than those without cirrhosis: septic shock and advanced liver disease (Child–Pugh class 3) were associated with increased mortality. Finally, Gustot *et al*.^23^ conducted a study to examine the epidemiology of infections in patients admitted to intensive care units. The prevalence of infection was higher in patients with cirrhosis than in those without. Gram-positive pathogens and MRSA were frequent and mortality was higher in cirrhotic compared with non-cirrhotic patients.

Our study confirmed that that MRSA carriage and bacteraemia were both more frequent in patients with advanced liver disease and associated with poor outcome, which is consistent with previous studies. These findings highlight the need to intensify MRSA prevention and control strategies in this vulnerable patient population. Options for MRSA control in high-risk patients include MRSA screening and decolonization in the pre-hospital or outpatient setting at an earlier stage in their illness, intensified MRSA screening in the inpatient setting followed by relevant control measures to prevent onwards transmission or vaccination of at-risk patients to prevent *S. aureus* disease (in the event that one becomes available). In the UK, MRSA decolonization is routinely undertaken using a standard regimen of an antibacterial nasal cream and body wash, as recommended by the Department of Health.^24^ However, patients with skin lesions and carriage in extranasal sites have a lower chance of clearing MRSA carriage with nasal mupirocin alone.^25^ In some countries such as the Netherlands, systemic antibiotics are added to the decolonization regimen of complicated cases (defined as MRSA infection, skin lesions, foreign-body material, mupirocin resistance and/or exclusive extranasal carriage).^26^ UK guidelines published by the BSAC also recommend the use of mupirocin with an oral antibiotic for treatment and clearance of mupirocin-susceptible MRSA, but acknowledge the limited evidence base for this recommendation.^22^ Indeed, a Cochrane systematic review of antimicrobial agents for treating MRSA colonization (which included 384 participants in six trials) found insufficient evidence to support the use of topical or systemic antimicrobial therapy for eradicating nasal or extranasal MRSA.^27^ There was no difference between topical and systemic therapy, or a combination of these agents, and concerns were raised about potentially serious adverse events or the development of antimicrobial resistance. Another strategy that could be considered in patients with chronic liver disease is vaccination against *S. aureus.* This has been evaluated in clinical trials for patients with end-stage renal failure undergoing haemodialysis,^28^ and undergoing cardiothoracic surgery,^29^ with limited efficacy. Reasons for the failure of current strategies for *S. aureus* vaccination relate to vaccine design, failure to elicit an appropriate functional and opsonophagocytic killing response and appropriate choice of target population.^30^

In response to these MRSA bacteraemia cases, the infection control team at CUH initiated intensified (fortnightly) MRSA screening of inpatients on the hepatology ward over the following 3 months after the last bacteraemia case, during which no new cases of MRSA colonization were identified on the ward. MRSA screening was also initiated in the hepatology outpatient clinic in an attempt to identify and decolonize patients prior to hospital admission with advanced liver disease.

A limitation of our study is that we only sequenced MRSA isolates from patients who were found to be MRSA carriers for the first time at CUH and had been admitted to the hepatology ward during the previous 12 months, thereby excluding existing carriers. This targeted approach was chosen because it was cost-effective yet enriched for MRSA acquisition on the hepatology ward and would be predicted to reveal an extended transmission network developing over time. The possibility remains, however, that new acquisitions resulted from transmission from long-standing carriers who were not sampled.

This study has highlighted some of the many challenges faced by clinicians managing nosocomial MRSA bacteraemia in complex, vulnerable patients such as those admitted to tertiary referral centres. Despite the existence of robust systems for MRSA control, targets for reductions in MRSA bacteraemia in English hospitals set by the Department of Health may not be met by specialist hospitals that receive complex patients who have had previous and often multiple admissions elsewhere and who are already colonized with MRSA at presentation. The point at which MRSA acquisition could be prevented has often passed and is beyond the control of the receiving referral centre. Although MRSA decolonization is an important principle in UK infection control practice, it will often fail in complex patients with defects in skin integrity or the presence of one or more medical devices such as intravascular or urinary catheters. UK hospitals face the imposition of harsh financial penalties when they exceed targets for MRSA bacteraemias and other nosocomial infections, but the evidence base upon which annual targets are set for a given facility is slim. We believe that the application of terms such as ‘zero tolerance’ to healthcare-associated infections is unrealistic in the context of events that may not be preventable. Nevertheless, CUH has made it clear that a key strategic aim of the hospital is to ensure that no avoidable infections occur. Additional research is required to develop more discriminatory assessment tools that provide a prediction of risk for progression to MRSA bacteraemia in MRSA carriers. Furthermore, it is necessary to take into account the contribution of preventable versus non-preventable risk factors in order to design a rational strategy for prevention of healthcare-associated infections. The imposition of punitive financial penalties for exceeding targets for healthcare-associated infections is unlikely to improve infection control practices or benefit patient care.

## Funding

This study was funded by grants from the UKCRC Translational Infection Research Initiative (MRC grant number G1000803), the Wellcome Trust (grant number 098051), Public Health England (grant number 107514) and the NIHR Cambridge Biomedical Research Centre. M. E. T. is a Health Foundation/Academy of Medical Sciences Clinician Scientist Fellow.

## Transparency declarations

M. E. T. has received support for conference travel and accommodation from Illumina Inc. N. M. B. is a consultant for Discuva Ltd. J. P. has received support for conference travel and accommodation from Pacific Biosciences of California Inc. and Illumina Inc. S. J. P. is a consultant for Pfizer Inc. All other authors: none to declare.

## Supplementary data

Table S1 is available as Supplementary data at *JAC* Online (http://jac.oxfordjournals.org/).

Supplementary Data
