# Supplementary material for: Zero tolerance for healthcare-associated MRSA bacteraemia: is it realistic?
Source: J Antimicrob Chemother. 2014 Apr 30;69(8):2238–45. doi: 10.1093/jac/dku128 (PMC4100711; doi:10.1093/jac/dku128)
Supplement: Supplementary Data [file supp_69_8_2238__index.html]

Zero tolerance for healthcare-associated MRSA bacteraemia: is it realistic? — Zero tolerance for healthcare-associated MRSA bacteraemia: is it realistic? — Supplementary Data 

# Zero tolerance for healthcare-associated MRSA bacteraemia: is it realistic?

## Supplementary Data

Supplementary Data

**Files in this Data Supplement:**

- Supplementary Data - Docx file
