# Supplementary material for: Zero tolerance for healthcare-associated MRSA bacteraemia: is it realistic?
Source: J Antimicrob Chemother. 2014 Apr 30;69(8):2238–45. doi: 10.1093/jac/dku128 (PMC4100711; doi:10.1093/jac/dku128)
Supplement: Supplementary Data [file supp_dku128_dku128supp.docx]

**Supplementary data**

**Table S1.** Patient number, isolate number, MLST and accession number of isolates included in the study

| **Patient number** | **Isolate number** | **MLST** | **Accession number** |
| --- | --- | --- | --- |
| P1 | 1A | 22 | ERR230478 |
| P1 | 1B | 22 | ERR230481 |
| P1 | 1C | 22 | ERR230476 |
| P2 | 2A | 2046 | ERR230474 |
| P2 | 2B | 2046 | ERR230480 |
| P3 | 3A | 22 | ERR230479 |
| P3 | 3B | 22 | ERR230475 |
| P4 | 4A | 22 | ERR230484 |
| P4 | 4B | 22 | ERR230482 |
| P4 | 4X | 22 | ERR156451 |
| P5 | 5 | 22 | ERR232591 |
| P6 | 6 | 22 | ERR232540 |
| P7 | 7 | 22 | ERR232559 |
| P8 | 8 | 22 | ERR232560 |
| P9 | 9 | 1 | ERR232542 |
| P10 | 10 | 22 | ERR232544 |
| P11 | 11 | 22 | ERR232546 |
| P12 | 12 | 22 | ERR232548 |
| P13 | 13 | 59 | ERR232551 |
| P14 | 14 | 8 | ERR232552 |
| P15 | 15 | 22 | ERR232553 |
| P16 | 16 | 22 | ERR232555 |
| P17 | 17 | 22 | ERR232557 |
| P18 | 18 | 22 | ERR232558 |
| P19 | 19 | 22 | ERR232562 |
| P20 | 20 | 36 | ERR232567 |
| P21 | 21 | 8 | ERR232568 |
| P22 | 22 | 22 | ERR232570 |
| P23 | 23 | 22 | ERR232572 |
| P24 | 24 | 22 | ERR232574 |
| P25 | 25 | 22 | ERR232575 |
| P26 | 26 | 22 | ERR232576 |
| P27 | 27 | 22 | ERR232577 |
| P28 | 28 | 22 | ERR232578 |
| P29 | 29 | 22 | ERR232579 |
| P30 | 30 | 22 | ERR232580 |
| P31 | 31 | 22 | ERR232583 |
| P32 | 32 | 22 | ERR232585 |
| P33 | 33 | 36 | ERR232586 |
| P34 | 34 | 22 | ERR232587 |
| P35 | 35 | 22 | ERR232588 |
| P36 | 36 | 22 | ERR232589 |
| P37 | 37 | 22 | ERR232590 |
| P38 | 38 | 22 | ERR232592 |
| P39 | 39 | 22 | ERR232593 |
| P40 | 40 | 22 | ERR232594 |
| P41 | 41 | 22 | ERR232595 |
| P42 | 42 | 72 | ERR232596 |
| P43 | 43 | 149 | ERR232597 |
| P44 | 44 | 22 | ERR232598 |
| P45 | 45 | 22 | ERR232599 |
| P46 | 46 | 1 | ERR232602 |
